# Supplementary material for: Genome-wide CRISPR Screens in T Helper Cells Reveal Pervasive Crosstalk between Activation and Differentiation
Source: Cell. 2019 Feb 7;176(4):882–896.e18. doi: 10.1016/j.cell.2018.11.044 (PMC6370901; doi:10.1016/j.cell.2018.11.044)
Supplement: Data S2. Processed Data from All the Steps of the Analysis, Related to Figure 1 [file mmc2.zip › supplemental data/motif analysis/Stat6_SRX021632_homer/homerResults/motif1.similar.html]

motif1

## Information for motif1

C
A
T
G
T
C
G
A
G
C
A
T
A
C
T
G
G
C
T
A
T
A
G
C
C
G
A
T
T
G
A
C
G
C
T
A
A
G
C
T
  
Reverse Opposite:  

T
C
G
A
C
G
A
T
A
C
T
G
G
C
T
A
A
T
C
G
C
G
A
T
T
G
A
C
C
G
T
A
A
G
C
T
G
T
A
C
  

|  |  |
| --- | --- |
| p-value: | 1e-2611 |
| log p-value: | -6.014e+03 |
| Information Content per bp: | 1.621 |
| Number of Target Sequences with motif | 6332.0 |
| Percentage of Target Sequences with motif | 25.05% |
| Number of Background Sequences with motif | 1142.8 |
| Percentage of Background Sequences with motif | 4.73% |
| Average Position of motif in Targets | 101.2 +/- 43.5bp |
| Average Position of motif in Background | 98.2 +/- 64.0bp |
| Strand Bias (log2 ratio + to - strand density) | -0.0 |
| Multiplicity (# of sites on avg that occur together) | 1.20 |
| Motif File: | file (matrix) reverse opposite |

### Similar de novo motifs found

|  |  |  |  |  |  |  |  |
| --- | --- | --- | --- | --- | --- | --- | --- |
| Rank | Match Score | Redundant Motif | P-value | log P-value | % of Targets | % of Background | Motif file |
| 1 | 0.988 | T C G A C A G T A C T G G C T A A T C G C G A T T G A C G C T A A G T C G A C T A G C T A G T C | 1e-2503 | -5765.041176 | 24.67% | 4.80% | motif file (matrix) |
| 2 | 0.973 | T C G A A C G T A C T G G C T A T A C G A C G T G T A C C G T A | 1e-2485 | -5722.619280 | 23.96% | 4.53% | motif file (matrix) |
| 3 | 0.879 | T G C A A G C T A C T G G C T A A G T C C G A T T G C A T C G A | 1e-2049 | -4719.335219 | 26.58% | 6.82% | motif file (matrix) |
| 4 | 0.918 | A G C T A C T G G C T A T A G C C G A T G T A C C T G A A T G C | 1e-1736 | -3999.121729 | 23.56% | 6.21% | motif file (matrix) |
| 5 | 0.753 | T C G A G C A T T A C G G C T A T G A C C G T A A T G C G T C A A C G T T A G C | 1e-512 | -1180.866056 | 13.20% | 5.19% | motif file (matrix) |
| 6 | 0.784 | A C G T A G C T T A G C A C G T C A T G C G T A T A G C A C G T C G T A T C G A | 1e-424 | -977.236327 | 9.10% | 3.17% | motif file (matrix) |
| 7 | 0.804 | G T C A C A T G T C G A C A G T A T C G T C G A A T G C C G A T C T G A G C T A C T A G G T C A | 1e-410 | -944.571699 | 7.89% | 2.55% | motif file (matrix) |
| 8 | 0.612 | A T G C G C A T C A T G T G C A C A G T T A C G G C T A G T A C C T A G A T G C T G C A T C G A | 1e-58 | -135.703957 | 0.19% | 0.01% | motif file (matrix) |
